# Global analysis of gene expression in NGF-deprived sympathetic neurons identifies molecular pathways associated with cell death

**DOI:** 10.1186/1471-2164-12-551

**Published:** 2011-11-08

**Authors:** Mark Kristiansen, Francesca Menghi, Rosie Hughes, Mike Hubank, Jonathan Ham

**Affiliations:** 1 Molecular Haematology and Cancer Biology Unit, Institute of Child Health, University College London, 30 Guilford Street, London WC1N 1EH, UK

## Abstract

**Background:**

Developing sympathetic neurons depend on nerve growth factor (NGF) for survival and die by apoptosis after NGF withdrawal. This process requires *de novo *gene expression but only a small number of genes induced by NGF deprivation have been identified so far, either by a candidate gene approach or in mRNA differential display experiments. This is partly because it is difficult to obtain large numbers of sympathetic neurons for *in vitro *studies. Here, we describe for the first time, how advances in gene microarray technology have allowed us to investigate the expression of all known genes in sympathetic neurons cultured in the presence and absence of NGF.

**Results:**

We have used Affymetrix Exon arrays to study the pattern of expression of all known genes in NGF-deprived sympathetic neurons. We identified 415 up- and 813 down-regulated genes, including most of the genes previously known to be regulated in this system. NGF withdrawal activates the mixed lineage kinase (MLK)-c-Jun N-terminal kinase (JNK)-c-Jun pathway which is required for NGF deprivation-induced death. By including a mixed lineage kinase (MLK) inhibitor, CEP-11004, in our experimental design we identified which of the genes induced after NGF withdrawal are potential targets of the MLK-JNK-c-Jun pathway. A detailed Gene Ontology and functional enrichment analysis also identified genetic pathways that are highly enriched and overrepresented amongst the genes expressed after NGF withdrawal. Five genes not previously studied in sympathetic neurons - *trib3, ddit3, txnip, ndrg1 *and *mxi1 *- were validated by real time-PCR. The proteins encoded by these genes also increased in level after NGF withdrawal and this increase was prevented by CEP-11004, suggesting that these genes are potential targets of the MLK-JNK-c-Jun pathway.

**Conclusions:**

The sympathetic neuron model is one of the best studied models of neuronal apoptosis. Overall, our microarray data gives a comprehensive overview of, and provides new information about, signalling pathways and transcription factors that are regulated by NGF withdrawal.

## Background

During normal nervous system development, neurons depend on growth factors secreted by their target tissues for survival [1]. These neurotrophic factors bind to cell surface receptors on developing neurons and activate intracellular signalling pathways that inhibit programmed cell death and promote neuronal growth. The regulation of programmed cell death by survival factors plays an integral part in ensuring that neuronal populations of the correct size are established [1-3]. In addition, increasing evidence suggests that apoptosis contributes to the neuronal loss seen after acute injuries to the nervous system, such as stroke or trauma, or in cell culture and animal models of neurodegenerative disorders, such as Parkinson's disease and Alzheimer's disease [4]. Developing sympathetic neurons have proved to be a valuable model for studying the molecular mechanisms of apoptosis and the signalling pathways that regulate neuronal death [5-11]. These cells require nerve growth factor (NGF) for survival during late embryonic and early postnatal development. When deprived of NGF, sympathetic neurons die by apoptosis and this death is inhibited by actinomycin D and cycloheximide suggesting that new gene expression is required for cell death to occur [12]. The key prediction of this hypothesis is that the transcription of specific genes increases after NGF withdrawal and that the proteins encoded by these induced genes trigger cell death.

To date only a limited number of induced genes that promote apoptosis have been identified, either by studying the expression of candidate genes (*c-jun, bim, tp63, puma*) or in mRNA differential display experiments (*egln3*). In the case of each of these genes the mRNA and protein increases in level after NGF withdrawal and experiments with knockout mice have demonstrated that the gene is required for NGF withdrawal-induced death [13-23]. However, the intracellular signalling pathways that are altered by NGF withdrawal - the MLK-JNK-c-Jun pathway is activated and the PI3K-Akt and Raf-MEK-ERK pathways are inactivated - are likely to regulate the expression of a much larger number of genes. Some of these genes, like *bim *and *puma*, will directly regulate the intrinsic pathway of caspase activation. However, other genes induced after NGF withdrawal may be involved in other aspects of NGF withdrawal-induced death, e.g. alterations in signalling pathways, changes in cell shape, the decrease in the rate of protein synthesis or neurite fragmentation. No previous study has comprehensively addressed these issues in sympathetic neurons. Recent advances in gene microarray technology have allowed us to investigate the expression of all known genes in sympathetic neurons for the first time. The Affymetrix Rat Exon 1.0ST microarray allows more accurate measurement of gene expression at the whole gene level because there are several oligonucleotide probes for each exon of a gene. In addition, exon arrays can be used to measure the expression of individual exons, which provides information about alternative splicing. Microarray analysis represents an unbiased approach to the investigation of NGF-withdrawal induced apoptosis and will identify the majority of the genes that are up- or down-regulated after NGF withdrawal.

Using developing sympathetic neurons as a model system, we have carried out a genome-wide analysis of gene expression at 16 hours following NGF withdrawal. Furthermore we have analysed gene expression in NGF-deprived sympathetic neurons in the presence or absence of the MLK inhibitor, CEP-11004 [24]. By including CEP-11004 in our experimental design we were able to identify which of the genes induced after NGF withdrawal are potential targets of the MLK-JNK-c-Jun signalling pathway, which is activated after NGF withdrawal and required for NGF deprivation-induced death [13,14,25-30]. To provide further insight into the molecular mechanisms that underlie NGF withdrawal-induced apoptosis in sympathetic neurons we also performed functional analysis that identified highly enriched genetic pathways. Our data provides a comprehensive overview of how NGF withdrawal alters signalling pathways and global gene expression. This will increase our understanding of the basic mechanisms of neuronal apoptosis and may also identify new targets for the development of neuroprotective drugs.

## Results

### Temporal analysis of NGF withdrawal induced apoptosis in sympathetic neurons

To comprehensively study the expression of all known genes in rat sympathetic neurons we used Affymetrix Exon arrays to profile gene expression at 16 hours after NGF withdrawal compared to +NGF as a control. We selected 16 hours because this was previously defined as the transcriptional commitment point [5] and induced genes known to be required for NGF withdrawal-induced death, e.g. *c-jun, bim, egln3*, are expressed at a high level at this time.

**Figure 1:**
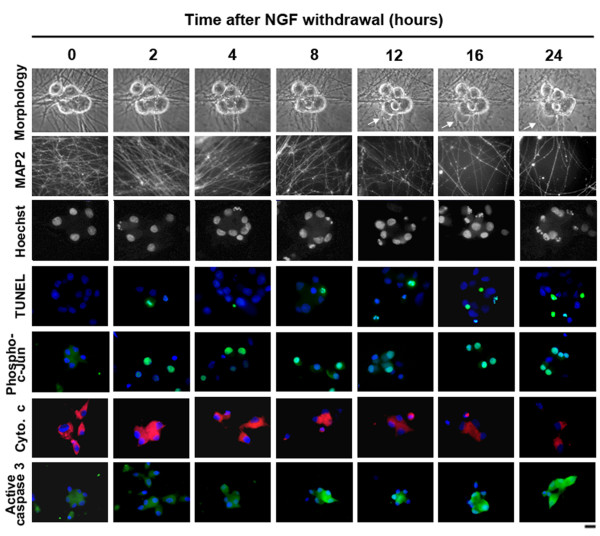
**Timecourse of NGF withdrawal-induced apoptosis in sympathetic neurons**. Rat sympathetic neurons were cultured in the presence of NGF for 6 days *in vitro *and then maintained in medium lacking NGF and containing anti-NGF antibody for the times indicated. Timelapse microscopy shows that sympathetic neurons develop an apoptotic morphology during the first 24 hours following NGF withdrawal with membrane blebbing (white arrows) and cell shrinkage occurring. Immunocytochemistry using a MAP2 antibody shows that between 4 and 8 hours after NGF withdrawal, neurites become fragmented and by 24 hours there are significantly fewer neurites compared to the control (0 hours). These morphological changes are accompanied by nuclear changes indicative of apoptosis visualised by Hoechst and TUNEL staining and these are seen as early as 2-4 hours after NGF withdrawal. Importantly, c-Jun becomes phosphorylated and is localised exclusively in the nucleus after NGF-withdrawal. Cytochrome c is released from the mitochondria and decreases in level. Finally, caspase-3 becomes activated and is clearly detected from 8 hours following NGF deprivation. The scale bar represents 20 μm.

To be able to relate any changes in gene expression that we might observe to the morphological and biochemical changes that are known to occur after NGF withdrawal we carried out a temporal analysis of NGF withdrawal-induced apoptosis using several well-defined markers (Figure 1). The morphological changes that occur in sympathetic neurons following NGF withdrawal are apparent after 8-12 hours of NGF deprivation (Figure 1). During this time, the smooth appearance of the plasma membrane is lost and the cell becomes irregular in structure. This is accompanied by beading of the neurites. At later timepoints (16-24 h), membrane blebbing and extensive neurite degeneration occur shortly before the neuron starts to lose its structural integrity. Nuclear changes such as chromatin condensation and nuclear shrinkage were visualised by staining with Hoechst dye and DNA fragmentation was detected by TUNEL labelling. These changes occur rapidly after NGF withdrawal but become much more apparent from 12-16 hours (Figure 1). Other key apoptotic events such as cytochrome c release from the mitochondria and the activation of caspase 3 were also measured (Figure 1). Cytochrome c is released from the mitochondria following NGF withdrawal and eventually decreases in level. Similarly, caspase 3 becomes activated and is clearly detected in sympathetic neurons deprived of NGF from 8 hours. We also detected an increase in c-Jun phosphorylation at serine 63 following NGF withdrawal (Figure 1). This site is phosphorylated by JNKs, which are activated after NGF deprivation. Importantly, the level of c-Jun phosphorylation increases before and peaks at 16 hours. Therefore at 16 hours, the timepoint chosen for our Exon microarray analysis, the MLK-JNK-c-Jun pathway has been activated in many neurons, and some cells in the population are already undergoing apoptosis.

### Gene expression profiling in sympathetic neurons after NGF withdrawal

**Figure 2:**
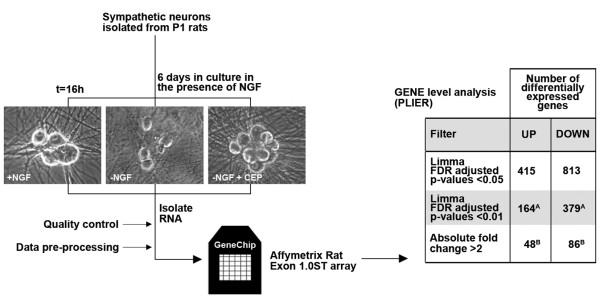
**Schematic overview of gene-level analysis**. Rat sympathetic neurons were cultured *in vitro *for 6 days and then maintained in 1) medium containing NGF, or 2) medium without NGF supplemented with anti-NGF antibody or 3) medium without NGF containing anti-NGF antibody and the MLK inhibitor CEP-11004 at 400 nM. Three independent experiments were performed. After 16 hours, RNA was isolated and processed and gene expression analysed using Affymetrix rat exon microarrays. The summarisation algorithm, PLIER (Probe Logarithmic Intensity Error) was used to compute gene-level expression values. At the gene level, we identified 1228 differentially expressed genes with a Limma FDR (false discovery rate) adjusted p-value of < 0.05. This number of differentially expressed genes was reduced to 48 up-regulated and 86 down-regulated genes when applying a more stringent statistical test (FDR adjusted p-value < 0.01) and an absolute fold change threshold of > 2. A - see additional file 1 for a list of these genes. B - see additional file 2 for a list of these genes.

To identify new genes that may play a role in NGF withdrawal-induced apoptosis, we performed a gene microarray analysis using Affymetrix Exon arrays and RNA isolated from sympathetic neurons that had been cultured for 16 hours in the presence of NGF (+NGF), absence of NGF (-NGF) or absence of NGF but with the MLK inhibitor CEP-11004 (-NGF+CEP-11004) added to the medium (Figure 2). MLKs are upstream activators of the JNK pathway in sympathetic neurons and CEP-11004 therefore blocks the increase in JNK activity and c-Jun phosphorylation and protects against NGF withdrawal-induced death. Three independent experiments were performed. Quality control and data analysis revealed good normalisation and reproducibility. An FDR-corrected p-value of 0.05 was used as an initial cut off to identify statistically significant differences in gene expression between each of the three different treatment groups (+NGF, -NGF, -NGF+CEP-11004). Each individual comparison generated a list of differentially expressed genes which were either up- or down-regulated in sympathetic neurons. When comparing the +NGF and -NGF treatment groups this analysis revealed 415 genes that were up-regulated and 813 genes that were down-regulated (Figure 2). A more stringent statistical threshold with an FDR-adjusted p-value of < 0.01 reduced this number to 164 and 379 up- and down-regulated genes respectively. Further analysis revealed that of the up-regulated genes with a FDR adjusted p-value of < 0.01, 48 genes had a fold change of greater than 2 (Additional file 1). Similarly, the expression of 86 of the genes that were down-regulated changed in level by greater than 2-fold (Additional file 2).

We also checked our microarray data for the genes previously shown to be regulated by NGF withdrawal in sympathetic neurons, such as *c-jun*, *dp5*, *bim*, *egln3 *and *cyclinD1 *and found that their expression had changed as predicted (Table 1; [13-17,24,31-35]). Importantly, the induction after NGF withdrawal of those genes previously defined as targets of the MLK-JNK-c-Jun pathway, *c-jun, bim, dp5, mkp1 *[17,19,24,26,34] was reduced by CEP-11004.

**Table 1:** Genes known to be regulated by NGF withdrawal

Gene symbols	Gene name	-NGF v +NGF	CEP v +NGF	Pathway	References
**Bid3/Dp5/Hrk**	BH3-interacting domain-containing protein 3; Dp5; harakiri	9.78	5.28	cell death	32, 34
**Trib3**	tribbles homologue 3 (Drosophila)	5.21	1.81	ER stress	35
**Dusp1/Mkp1**	dual specificity phosphatase 1;MAP kinase phosphatase 1	4.69	1.60	MAPK pathways	13, 24
**Bcl2l11/Bim/Bod**	BCL2-like 11 (apoptosis facilitator)	4.35	1.95	cell death	16, 17
**Egln3/SM-20**	EGL nine homologue 3 (C. elegans)	3.41	3.53	cell death	15
**Gadd45γ**	growth arrest and DNA-damage- inducible protein 45 gamma	3.16	1.31	DNA damage inducible	33
**Jun**	Jun oncogene; c-Jun	2.62	1.55	AP1 family	13, 14
**Ccnd1**	cyclin D1	1.46	1.04	cell cycle	31
**Myc**	myelocytomatosis viral oncogene homologue (avian); c-Myc	-1.76	-1.32	Myc network	31
**Ngfr/p75^NTR^**	nerve growth factor receptor (TNFR superfamily, member 16); p75^NTR^	-1.73	-1.44	neurotrophin signalling	31

The fold increase in RNA level after NGF withdrawal or after NGF withdrawal in the presence of CEP-11004 (average of 3 independent experiments) from the microarray is shown for genes that have been previously reported to be transcriptionally regulated in this system (see references above).

### Gene Ontology analysis and unsupervised hierarchical clustering

**Figure 3:**
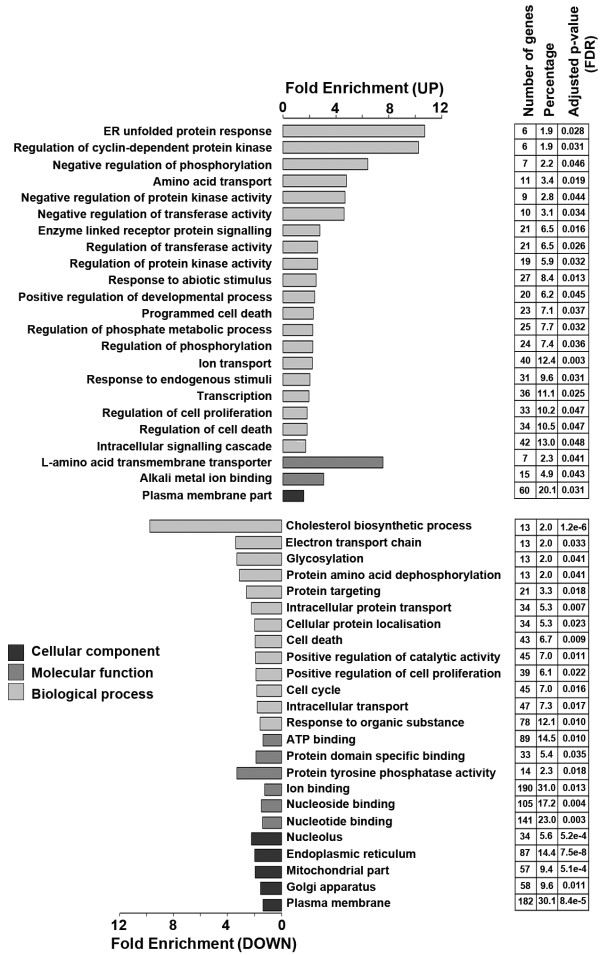
**Functional enrichment analysis of gene ontology terms**. Gene Ontology (GO)-based annotation was used to perform functional enrichment analysis using the DAVID (v6.7) tools. Fold enrichment of NGF-withdrawal regulated genes is measured by the bar length. The number of genes refers to the number of differentially expressed genes described by that annotation and is also expressed as a percentage of the total genes described for that term. An FDR adjusted p-value represents the significance of the enrichment. The key represents the three domains covered within the Gene Ontology. Only annotations with a significant FDR adjusted p-value of < 0.05 are shown.

Gene Ontology covers three domains: cellular component, molecular function and biological process. However, to obtain an overview of the functional categories and the biological relevance of the genes regulated by NGF withdrawal, we used an alternative strategy of functional analysis (rather than the standard Gene Ontology analysis). Functional enrichment analysis of Gene Ontology terms using DAVID identified those annotations that were significantly overrepresented amongst the list of genes significantly de-regulated after NGF withdrawal compared to a reference gene list consisting of all of the annotated genes represented on the microarray. All genes significantly up- or down-regulated (FDR = 0.05) after NGF withdrawal (1228 in total) were eligible for this analysis. Gene lists that contained up- or down-regulated genes were analysed separately. Following NGF withdrawal, major functional categories that were especially up-regulated included *intracellular signalling cascade*, *ion transport *and *transcription *(Figure 3). In contrast, the down-regulated genes appeared to be involved in cell cycle and intracellular transport as well as nucleoside and ion binding. Interestingly, twice as many genes associated with the cell death process were down-regulated compared to up-regulated. Functional categories such as the *ER unfolded protein response *and *negative regulation of protein kinase activity *were significantly over-represented in the up-regulated genes whilst in the down-regulated genes, categories such as *cholesterol biosynthetic process *and the *electron transport chain *were significantly enriched (Figure 3). Notably, the proportion of up-regulated genes related to *amino acid transport *and *positive regulation of developmental process *was also significantly higher than expected by chance. Finally, a significant proportion of genes associated with the cellular components plasma membrane, mitochondria and the ER was strikingly different between the up- and down-regulated genes (Figure 3).

**Figure 4:**
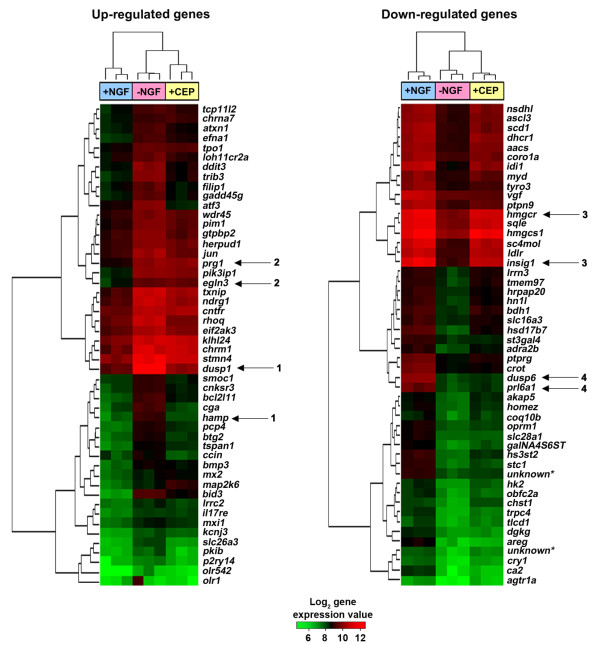
**Top 50 genes up- and down-regulated in sympathetic neurons after NGF withdrawal**. Heatmaps showing the expression of the 50 genes most significantly up- and down-regulated in sympathetic neurons after NGF withdrawal compared to other treatments as indicated. Cluster analysis was performed on the genes and the treatment groups according to their expression profiles and the corresponding dendrogram is shown to the left and to the top of the heatmap respectively. Treatment groups are colour coded as follows: blue, sympathetic neurons cultured in the presence of NGF for 16 hours (+NGF); pink, sympathetic neurons cultured in the absence of NGF and in the presence of an anti-NGF antibody (-NGF); yellow, sympathetic neurons cultured in the absence of NGF and in the presence of an anti-NGF antibody and the compound CEP-11004 (+CEP). A green-red colour palette illustrates the log_2 _gene expression values, with green corresponding to low and red corresponding to high expression. The numbered arrows refer to the 4 major patterns of expression as described in the results. *Genes with unknown annotation.

The 50 most significantly up- and down-regulated genes were subjected to hierarchical clustering analysis (Figure 4). Both samples and genes were clustered according to their expression profiles using the Euclidean distance metric to calculate dendrograms. Genes with similar expression profiles may be regulated by common pathways and be involved in related functions. We observed four major patterns of gene expression (Figure 4): 1) genes induced after NGF withdrawal that require the MLK-JNK pathway, for example *dusp1 *and *hamp *(their induction was reduced by CEP-11004); 2) genes induced after NGF withdrawal that may not depend on the MLK-JNK pathway, e.g. *egln3 *and *prg1*; 3) genes that are down-regulated after NGF withdrawal that are rescued by CEP-11004, e.g. *hmgcr *and *insig1*, and 4) genes that are down-regulated after NGF withdrawal whose decrease in expression is not affected by CEP-11004, e.g. *dusp6 *and *prl6a1*. Genes belonging to some of the most common networks are listed in Table 2.

**Table 2:** Selection of genes regulated by NGF withdrawal identified in this study

GO pathway	Gene symbol	Gene name	-NGF v +NGF	CEP v +NGF
**AP1/ATF family**	**CREM**	cAMP responsive element modulator	1.51	1.25
	**JunD**	Jun D proto-onocogene	1.74	1.60
**Cell death**	**Bmf**	Bcl2 modifying factor	1.91	1.31
	**Casp12**	caspase 12	1.46	1.23
	**Casp3**	caspase 3, apoptosis related cysteine protease	1.73	1.31
	**Casp4**	caspase 4, apoptosis related cysteine protease	1.60	1.03
	**Cycs**	cytochrome c, somatic	-1.88	-1.19
**DNA damage-inducible**	**GADD45α**	Growth arrest and DNA damage-inducible 45 alpha	1.45	1.41
**ER stress**	**ATF3**	activating transcription factor 3	2.89	-1.69
	**Ddit3**	DNA damage-inducible transcript 3/CHOP10	4.11	1.37
	**Eif2ak3**	eukaryotic translation initiation factor 2 alpha kinase 3/PERK	2.28	1.54
	**Herpud1**	homocysteine-inducible, endoplasmic reticulum stress-inducible, ubiquitin-like domain member 1	2.17	1.17
	**Myd116**	myeloid differentiation primary response gene 116	1.81	1.09
**Fatty acid/cholesterol metabolism**	**Hmgcr**	3-hydroxy-3-methylglutaryl-coenzyme A reductase	-2.79	-1.76
	**Insig1**	insulin induced gene 1	-4.69	-1.51
	**Sc4mol**	sterol-C4-methyl oxidase-like	-3.95	-1.36
**FOXO target**	**Txnip**	thioredoxin interacting protein/Vdup1	3.68	2.46
**MAPK pathways**	**Dusp6**	dual specificity phosphatase 6/Mkp3	-6.68	-5.66
	**RhoQ**	Ras homologue gene family, member Q	2.51	1.16
**Miscellaneous**	**Areg**	amphiregulin	-5.90	-5.50
	**Bhlhb2**	basic helix-loop-helix domain containing, class B2	-2.35	-2.36
	**Btg1**	B-cell translocation gene 1, anti-proliferative	1.78	1.44
	**Btg2**	B-cell translocation gene 2, anti-proliferative	3.63	1.64
	**Chrna7**	cholinergic receptor, nicotinic, alpha polypeptide 7	2.16	2.16
	**Cnskr3**	Cnksr family member 3 (Magi1)	3.14	1.54
	**Pkib**	protein kinase (cAMP-dependent, catalytic) inhibitor beta	2.39	1.02
	**Tpo1**	developmentally regulated protein TPO1	2.41	2.04
**Myc network**	**Id2**	Inhibitor of DNA binding 2	-1.75	-1.31
	**Mxi1**	Max interacting protein 1	2.22	1.77
	**Mycn**	v-myc myelocytomatosis viral related oncogene, neuroblastoma derived	-2.89	-1.87
	**Ndrg1**	N-myc downstream regulated gene 1	3.18	2.07
	**Ptma**	prothymosin alpha	-1.93	-1.37
**PI3K-Akt**	**PIK3IP1**	similar to HGFL protein	5.28	4.56
**pathway**	**PIK3r1**	phosphatidylinositol 3-kinase, regulatory subunit, polypeptide 1/p85	1.85	1.90

The fold increase in RNA level after NGF withdrawal or after NGF withdrawal in the presence of CEP-11004 (average of 3 independent experiments) is shown for a selection of genes that have been grouped according to functional activity. The level of gene expression in the presence of NGF was set to 1. Negative fold changes refer to down-regulation. Only genes with a FDR-adjusted p-value of < 0.01 are shown.

### Validation of gene induction after NGF withdrawal by real time PCR

**Figure 5:**
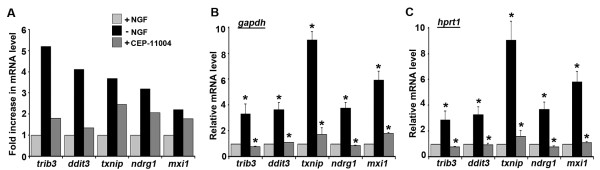
**Validation of gene induction after NGF withdrawal by real time PCR**. Rat sympathetic neurons were cultured *in vitro *for 6 days and then maintained for 16 hours in 1) medium containing NGF, or 2) medium without NGF supplemented with anti-NGF antibody or 3) medium without NGF containing anti-NGF antibody and the compound CEP-11004 at 400 nM. RNA was then isolated using an RNeasy kit. The experiment was performed in triplicate and the resulting RNA samples were then processed and an Affymetrix rat exon microarray analysis was carried out. *A*, The fold increase in RNA level after NGF withdrawal or after NGF withdrawal in the presence of CEP-11004 determined by exon array analysis (average of 3 independent experiments) is shown for 5 representative genes. For each gene, the level of expression in the presence of NGF was set as 1 and the expression levels for the other treatments were normalised relative to this. *B-C*, mRNA levels for the 5 genes were also measured by real time PCR using RNA prepared from three independent sets of neurons and normalised to the housekeeping genes *gapdh *(B) or *hprt1 *(C). The data shown represents the average of three independent experiments ± SEM. Statistical comparisons were made between +NGF and -NGF or -NGF and +CEP-11004 for each gene. *p < 0.01.

Functional enrichment analysis revealed that the annotation ER-unfolded protein response was the most enriched term after NGF withdrawal suggesting that an ER stress response occurs in sympathetic neurons deprived of NGF. We therefore initially selected two regulated genes from this category for further validation. *Trib3 *(up-regulated 5.2-fold after NGF withdrawal) and *ddit3/CHOP10 *(up-regulated 4.11-fold) were the third and ninth most up-regulated genes respectively after NGF withdrawal (Figure 5A). The *trib3 *mRNA was previously shown to increase in level after NGF withdrawal in PC12 cells [35] but nothing is known about its role in sympathetic neurons. CHOP10 has not been studied before in sympathetic neurons. The increase in the level of the *trib3 *and *ddit3/chop10 *mRNAs was reduced by CEP-11004, suggesting that these genes are potential targets of the MLK-JNK-c-Jun pathway. To validate these exon array results, we cultured sympathetic neurons for 6 days in the presence of NGF and then for a further 16 hours in the presence or absence of NGF ± CEP-11004. The levels of *trib3 *and *ddit3 *mRNA were then measured by quantitative real time PCR (Figures 5B-C). After NGF withdrawal, the levels of *trib3 *mRNA and *ddit3 *mRNA increased by 3.33-fold and 3.68-fold respectively but this was reduced to 0.79-fold and 1.1-fold in the presence of CEP-11004 when normalised to *gapdh *(Figure 5B). A similar increase was seen in *trib3 *and *ddit3 *mRNA levels after NGF withdrawal when normalised to *hprt1 *(Figure 5C). We also found that the *txnip *gene was significantly up-regulated after NGF withdrawal. Txnip binds to and inhibits thioredoxin, a major antioxidant protein in neurons. Any perturbation of the redox system in neurons could lead to a cellular pro-oxidant state that is a necessary component of apoptotic death [36]. We found that the *txnip *mRNA levels mirrored the patterns from microarray analysis (Figure 5). Interestingly, *txnip *mRNA levels increased significantly after NGF withdrawal (up-regulated 9.03-fold) and this was reduced to 1.73-fold in the presence of CEP-11004 when measured by qPCR and normalised to either *gapdh *or *hprt1*. Two other genes were also validated by quantitative PCR: *ndrg1 *and *mxi1*. Both of these genes are associated with the Myc gene regulation network and are induced after NGF withdrawal by 3.18-fold and 2.22-fold respectively. Quantitative PCR confirmed the increase in mRNA levels for both of these genes (Figure 5A-C).

### The protein levels of selected regulated genes increase after NGF withdrawal

**Figure 6:**
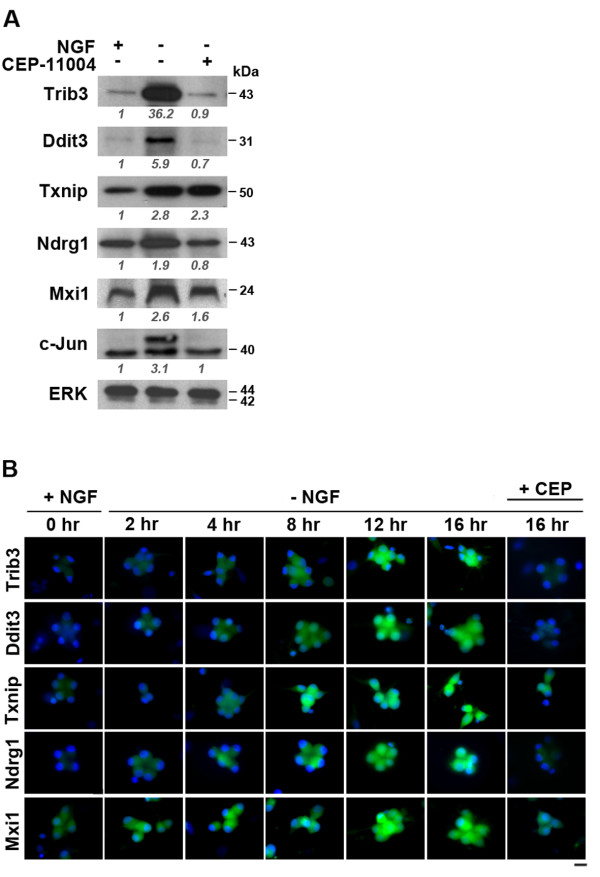
**The proteins encoded by the 5 selected genes increase in level after NGF withdrawal**. *A*, Immunoblotting analysis of Trib3, Ddit3, Txnip, Ndrg1 and Mxi1 protein levels using extracts prepared from sympathetic neurons cultured in the presence or absence of NGF ± CEP-11004 for 16 hours. For all 5 genes, protein levels were significantly increased at 16 hours after NGF withdrawal. Representative images are shown. c-Jun is shown as a positive control. ERK levels are shown as a loading control. The blots were scanned on a densitometer to quantitate the levels of each protein and were normalised to the ERK loading control. The ratio of the level of each protein in the presence of NGF (+NGF) to the level of each protein after NGF withdrawal (-NGF) or in the presence of CEP-11004 was then calculated. *B*, Analysis of Trib3, Ddit3, Txnip, Ndrg1 and Mxi1 protein levels and subcellular localisation by immunocytochemistry. Sympathetic neurons were treated as indicated and fixed at 0, 2, 4, 8, 12, or 16 hours after NGF withdrawal ± CEP-11004. The cells were then stained with the appropriate primary antibody followed by a FITC-conjugated secondary antibody, and Hoechst dye to label the nuclear DNA. Images were collected using the same exposure time for each timepoint and the coverslips in each gene group were analysed in parallel. The scale bar represents 20 μm.

We examined the effect of NGF withdrawal on the levels of the proteins encoded by the 5 selected genes and their localisation. In immunoblotting experiments, we observed a significant increase in the levels of the Trib3 and Ddit3 proteins by 16 hours after NGF withdrawal. In contrast, when sympathetic neurons were deprived of NGF in the presence of 400 nM CEP-11004 for 16 hours, there was no significant increase in the levels of these proteins when compared to neurons cultured in the presence of NGF (Figure 6A). Levels of Trib3 and Ddit3 protein and their subcellular localisation were also studied by immunofluorescence (Figure 6B). In the presence of NGF, Trib3 levels were low and levels of Ddit3 were almost undetectable. Levels of these proteins started to increase after ~8 hours peaking at 12-16 hours after NGF withdrawal. Trib3 was localised in both the nucleus and cytoplasm, whereas Ddit3 was localised mainly in the nucleus after NGF withdrawal. However, in the presence of CEP-11004, the levels of both proteins were decreased significantly to almost basal levels and more importantly were not detected in the nucleus. The protein levels of the other three genes: Txnip, Ndrg1 and Mxi1 were also studied by immunoblotting and immunofluorescence (Figure 6). Significant but modest increases in the levels of the Txnip, Ndrg1 and Mxi1 proteins were seen after NGF withdrawal and CEP-11004 reduced this to varying degrees. The increase in Txnip protein level after NGF withdrawal was smaller than that seen at the transcriptional level. The effect of CEP-11004 was also not as significant at the protein level. The increase in the level of the Txnip protein and its localisation after NGF withdrawal were also studied by immunofluorescence. The Txnip protein was clearly seen at 8 hours after NGF withdrawal in both the nucleus and cytoplasm and this was followed by a steady increase in protein levels over time. Both of the Myc pathway-associated proteins, Ndrg1 and Mxi1, also increased in level after NGF withdrawal and CEP-11004 reduced this increase (Figure 6).

### The *txnip *and *trib3 *promoters contain potential c-Jun binding sites

**Figure 7:**
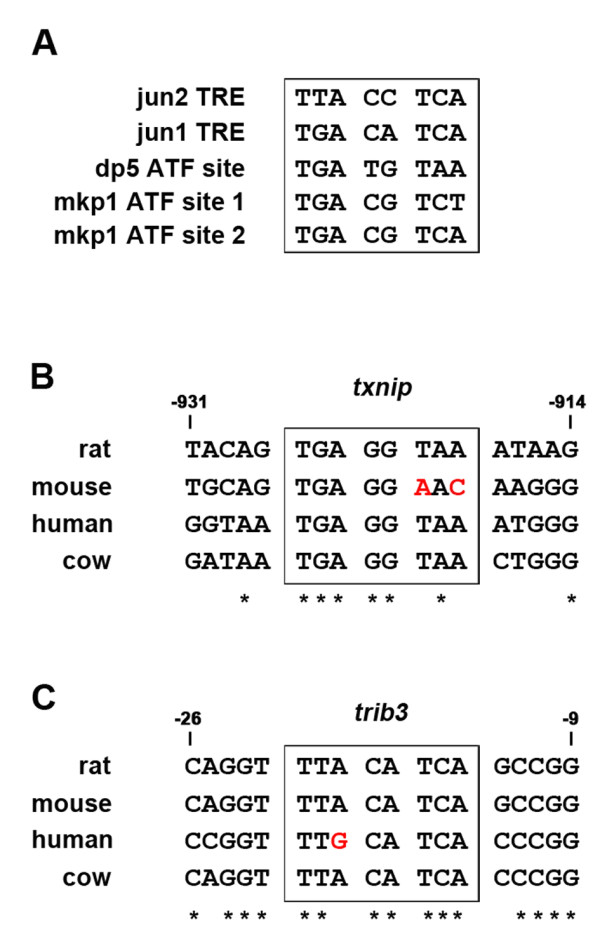
**The *txnip *and *trib3 *promoters contain potential c-Jun binding sites**. *A*, DNA sequences of functionally important ATF sites known to bind c-Jun and ATF2 in the *c-jun*, *dp5 *and *mkp1 *promoters. The *c-jun *promoter contains two ATF sites, the jun2 and jun1 TREs, the *dp5 *promoter contains one site, and the *mkp1 *promoter contains two sites. Each of these sites has been shown to bind c-Jun and ATF2 in ChIP assays and to be important for promoter induction after NGF withdrawal [24,26,34]. The orientation of the sequences is 5' to 3'. *B*, A conserved ATF site was identified in the *txnip *promoter using CONSITE software (http://asp.ii.uib.no:8090/cgi-bin/CONSITE/consite) to analyse orthologous pairs of genomic sequences. This sequence (5'-TGAGGTAA-3') is identical to the reverse complement of the jun2 TRE (5'-TTACCTCA-3') in the *c-jun *promoter. Numbers refer to position in relation to Exon 1 of the rat *txnip *gene, as defined in the NCBI entry for rat *txnip *(http://www.ncbi.nlm.nih.gov/gene/117514). * indicates nucleotides conserved in all four species. Two non-conserved nucleotides in the mouse ATF site are shown in red. *C*, A conserved ATF site was identified in the *trib3 *promoter using CONSITE software to analyse orthologous pairs of genomic sequences. This sequence (5'-TTACATCA-3') is identical to the reverse complement of the ATF site in the *dp5 *promoter. Numbers refer to position in relation to Exon 1 of the rat *trib3 *gene, as defined in the NCBI entry for rat *trib3 *(http://www.ncbi.nlm.nih.gov/gene/246273). * indicates nucleotides conserved in all four species. One non-conserved nucleotide in the human ATF site is shown in red.

We previously showed that three of the genes that are induced after NGF withdrawal in sympathetic neurons, *c-jun*, *dp5 *and *mkp1*, are direct targets of c-Jun [24,26,34]. The induction of these genes after NGF deprivation is strongly reduced by CEP-11004 and the *c-jun*, *dp5 *and *mkp1 *promoters contain functionally important ATF sites (Figure 7A) that have been shown to bind c-Jun/ATF2 heterodimers in chromatin immunoprecipitation assays and EMSA experiments [24,34]. Some of the induced genes identified in our exon array analysis might also be direct targets of c-Jun, in particular those whose mRNA induction after NGF withdrawal is strongly suppressed by CEP-11004, for example *txnip *and *trib3 *(Figure 5B and 5C). We therefore searched for conserved potential c-Jun binding sites in the promoter, first exon and first intron of the rat *txnip *and *trib3 *genes (Figure 7B and 7C). The *txnip *promoter contains an ATF site (TGAGGTAA), 919 bp upstream of Exon 1 in the rat gene, that is identical in sequence to the reverse complement of the jun2 TRE site in the *c-jun *promoter (Figure 7A and 7B). This site is conserved in the rat, human, and cow *txnip *genes and contains two base changes in the mouse gene (Figure 7B). In the case of *trib3*, we identified a conserved ATF site (TTACATCA) 14 bp upstream of Exon 1 in the rat gene (Figure 7C). This site is identical to the reverse complement of the ATF site in the *dp5 *promoter (Figure 7A) and is conserved in the rat, mouse and cow genes and only one nucleotide differs in the human *trib3 *gene (Figure 7C). The presence of these potential c-Jun/ATF2 binding sites in the promoters of the rat *txnip *and *trib3 *genes suggests that these genes might be direct targets of the MLK-JNK-c-Jun pathway.

## Discussion

In 1988, it was first proposed by Martin *et al. *[12] that new RNA and protein synthesis is required for NGF withdrawal-induced apoptosis in sympathetic neurons. However, since then only a small number of genes have been shown to be regulated in this system and these were identified either by candidate gene approaches or the differential display technique. This is partly because it is difficult to obtain large numbers of sympathetic neurons for *in vitro *studies. However, advances in technology have now allowed us to identify the majority of the genes regulated by NGF withdrawal in sympathetic neurons. Using Affymetrix exon arrays and RNA isolated from rat sympathetic neurons, we investigated the global pattern of gene expression at 16 hours after NGF withdrawal. This time-point represents the transcriptional commitment point for sympathetic neurons undergoing NGF withdrawal-induced apoptosis and induced genes known to be required for NGF withdrawal-induced death, e.g. *c-jun, bim*, and *egln3*, are expressed at a high level at this time. We were able to detect almost all of the genes known to be regulated after NGF withdrawal (Table 1) indicating the reliability of the microarray data. However, one exception was the previously described up-regulated gene *puma *[19] which is required for NGF withdrawal-induced death [21]. On further investigation, we found that no probe sets matching the *puma *gene were represented on the rat Affymetrix exon 1.0ST microarray. Nevertheless, microarray technology remains a reliable tool and represents the best method for obtaining a complete overview of patterns of gene expression in this system. In addition, microarray studies can identify candidate genes for functional studies. For example, in the microarray experiments described in this paper we identified *mkp1 *as a gene induced after NGF withdrawal that could be a target of the MLK-JNK-c-Jun pathway. We subsequently showed that *mkp1 *is a direct transcriptional target of the MLK-JNK-c-Jun pathway in sympathetic neurons and an important regulator of JNK activity and the rate of NGF withdrawal-induced death [24]. Microarrays have previously been used to study gene expression in potassium-deprived cerebellar granule neurons undergoing apoptosis [37]. The most highly up-regulated gene in this study, *trim17*, was subsequently shown to encode a novel E3-ubiquitin ligase that can initiate neuronal apoptosis in several *in vitro *models of transcription-dependent apoptosis, including cerebellar granule neurons and NGF-deprived sympathetic neurons [38].

Approximately 95% of the genes identified in our microarray study have never been shown before to be transcriptionally regulated during NGF withdrawal-induced apoptosis. We have been able to identify potential targets of the MLK-JNK-c-Jun pathway by including CEP-11004 in our experimental design. We selected 16 hours as the optimal time-point for our microarray study and therefore identifying those genes and pathways that define the dying neurons at this time point could add to our understanding of the molecular mechanisms involved in the neuronal death programme.

In our data set, we identified 164 genes that were significantly up-regulated (FDR, p < 0.01) after NGF withdrawal and the expression of 48 of these genes increased by more than 2-fold. Conversely, 379 genes were down-regulated when the significance threshold was set at p = 0.01 and the expression of 86 of these genes decreased by 2-fold or more. We performed Gene Ontology (GO) analysis and functional enrichment analysis to identify specific annotations that were enriched following NGF withdrawal. Whilst this type of analysis depends upon a controlled vocabulary and therefore has its limitations, it also represents a powerful method for extracting potentially useful biological information from our gene expression data.

In an analysis of transcription-dependent neuronal apoptosis proceeding via the mitochondrial pathway, functional categories such as intracellular signaling cascades, transcription and mitochondrial changes might be expected to be enriched. Whilst these categories are indeed enriched after NGF withdrawal, other categories that contain genes which could suggest additional hypotheses about the mechanisms of neuronal death were also highlighted. The significance of the induction of ER stress-associated genes, for example, may offer new insights into the cell death process, especially since a similar response was observed in cerebellar granule neurons undergoing apoptosis [37] and experiments in other systems suggest a role for interactions between the mitochondria and the ER. On the other hand, the down-regulation of genes associated with cholesterol and fatty acid biosynthesis may be associated with an inhibition of cell growth since cholesterol and fatty acids are required for the synthesis of membranes.

Cluster analysis allowed us to group the genes according to their pattern of expression, especially in the presence of the MLK inhibitor, CEP-11004. The expression of many of the genes induced after NGF withdrawal is reduced by CEP-11004, suggesting that they may be targets of the MLK-JNK-c-Jun pathway. This group includes *c-jun*, *dp5 *and *mkp1 *whose promoters contain ATF sites that bind c-Jun and which are important for their induction after NGF withdrawal (Figure 7A) [24,26,34]. The induction of a few genes, such as *egln3*, is not affected by CEP-11004, suggesting that the transcription of these genes may be regulated by other transcription factors that are activated after NGF withdrawal, but not regulated by the JNK pathway, for example, FOXO3a or Myb. Interestingly, CEP-11004 reverses the decrease in the level of expression of some of the genes that are down-regulated after NGF withdrawal. Many of these genes encode proteins involved in fatty acid metabolism and cholesterol metabolism, e.g. *insig1*, *sqle*, *hmgcr*, and *hmgcs1*, and their transcription is activated by sterol regulatory element-binding proteins (SREBPs). In sympathetic neurons, the MLK-JNK-c-Jun pathway may negatively regulate the activity of a key transcription factor or signaling protein that is important for the transcription of this set of genes.

The expression of only a small number of cell death genes changes after NGF withdrawal. *Bim*, *dp5*, and *puma *mRNA levels have been previously shown to increase after NGF deprivation and in this study we have confirmed this for *bim *and *dp5*. We also found that the *bmf*, *caspase-12*, *caspase-3*, and *caspase-4 *mRNAs increase in level whereas the expression of *cytochrome c *and *prothymosin alpha *(*ptma; *an inhibitor of the apoptosome; [39]) decreases after NGF withdrawal (Table 2). Thus in sympathetic neurons, as previously described for cerebellar granule neurons [37], the expression of the components of the intrinsic pathway (*bax*, *cytochrome c*, *apaf-1*, *caspase-9*), which are all essential for cell death, is not greatly altered by NGF withdrawal. However, what does change significantly is the level of expression of four genes that encode BH3-only proteins that activate the intrinsic pathway: *dp5*, *bim*, *bmf *(Tables 1 and 2) and *puma *[19].

NGF-deprived sympathetic neurons undergo several biochemical and morphological changes before committing to the neuronal death programme and some of these are likely to play an important role in triggering apoptosis. Interestingly, levels of mitochondrial-produced reactive oxygen species (ROS) are known to increase early after NGF withdrawal [40] and this causes a cellular pro-oxidant state which appears to be required for the release of cytochrome c [40]. The regulation of cellular redox balance is critically determined by the activity of several antioxidant systems [41,42] one of which is the thioredoxin system [42]. Thioredoxin itself is regulated by an endogenous inhibitor, Txnip [43,44] and a reduction in thioredoxin activity due to an increase in Txnip levels might lead to increased oxidation of thiol groups in cellular proteins and ultimately an increase in apoptosis. We found a 9-fold increase in the level of the *txnip *mRNA after NGF withdrawal and this was reduced to 1.73-fold in the presence of CEP-11004 (Figure 5) which was confirmed in NGF-dependent differentiated PC6-3 cells (data not shown). Importantly, the level of Txnip protein also increased significantly after NGF withdrawal and this increase was prevented by CEP-11004 (Figure 6). These data suggest that *txnip *is a potential target of the MLK-JNK-c-Jun pathway and may play an important role in triggering the apoptotic programme after NGF withdrawal.

The endoplasmic reticulum (ER) plays a significant role in how cellular proteins are processed, folded, modified and transported. In neurodegenerative diseases, these cellular processes may go wrong leading to various levels of ER stress that may contribute to neuronal death [45]. When sympathetic neurons are treated with the ER-stressor, tunicamycin, c-Jun becomes phosphorylated but this can be prevented using CEP-11004 [46]. Bcl-2 and Bcl-x_L _have been found to associate with both the mitochondrial outer membrane and the ER membrane and it has been reported that crosstalk can occur between the ER and the mitochondria in cells undergoing apoptosis [47,48]. We found that two of the most up-regulated genes after NGF withdrawal, *trib3 *and *ddit3*, are associated with the ER-unfolded protein response and CEP-11004 prevented their increase in expression suggesting that they are potential MLK-JNK-c-Jun targets (Figure 5). Furthermore, functional analysis revealed that the ER unfolded protein response annotation was the most overrepresented gene category after NGF withdrawal suggesting that an ER stress response occurs in sympathetic neurons under these conditions. The exact role of these genes in ER stress-induced apoptosis remains unclear, however, it has been shown that CHOP10, a known AP-1 target gene, is induced by both ER stress and oxidative stress [49]. A propapoptotic role for CHOP10 has been reported since its overexpression can lead to apoptosis [50], whilst MEFs derived from CHOP10-/- mice are resistant to ER stress-induced cell death [51]. However, the mechanism by which CHOP induces apoptosis still remains unclear. It has been shown that CHOP-induced cell death is associated with the translocation of Bax from the cytosol to the mitochondria and that CHOP-induced cell death can be prevented by the overexpression of Bcl-2 or the knockdown of Bax [52]. The link between CHOP and Bax translocation could involve a novel ER-stress inducible gene, *trib3*. It has been shown that *trib3 *is induced via the ATF4-CHOP pathway through the identification of a CHOP-binding site in the proximal portion of the promoter [53]. Also, ER stress can activate *bim *through CHOP-C/EBPα dependent transcriptional activation [54] and in other studies CHOP has been found to bind to the promoter of the proapoptotic Bcl-2 family member *puma *[55]. The relationship between ER stress, CHOP, Trib3 and BH3-only proteins may suggest an important role in the apoptotic pathway after NGF withdrawal. Furthermore, we also identified a conserved ATF site (TTACATCA) 14 bp upstream of Exon 1 in the rat *trib3 *gene (Figure 7C) which is identical to the reverse complement of the ATF site in the *dp5 *promoter. This potential c-Jun/ATF2 binding site in the promoter of the rat *trib3 *gene suggests that this gene might also be a direct target of the MLK-JNK-c-Jun pathway. We also found evidence of an increase in Trib3 and Ddit3/CHOP10 protein levels after NGF withdrawal and this increase was prevented by CEP-11004 (Figure 6).

NGF withdrawal leads to a decrease in PI3K and Akt activity resulting in FOXO activation. FOXO3a translocates into the nucleus [56,57] and has been shown to trigger apoptosis by activating the transcription of genes necessary for cell death, such as *bim*. The *mxi1 *gene is also a target of FOXO3a, which binds to sites in the first intron downstream of the Mxi1-SRα promoter [58]. Mxi1 dimerises with Max and binds to E-boxes and represses c-Myc and MycN target genes by recruiting co-repressors to their promoters [59]. Interestingly, the *mxi1 *mRNA increases in level after NGF withdrawal (Figure 5) whereas *c-myc *and *mycn *mRNA levels decrease (Tables 1 and 2). Overexpression of MycN in sympathetic neurons has been shown to partially protect against NGF withdrawal-induced death [60], so by antagonising MycN, Mxi1 might have a proapoptotic role in this system. Furthermore, the expression of genes that are activated by c-Myc and MycN decreases after NGF deprivation, for example *id2 *and *ptma *(Table 2). *Ptma *can act as a repressor of the apoptosome [39] so it will be interesting to determine whether Ptma protein levels also decrease after NGF withdrawal. Conversely, the expression of genes repressed by c-Myc and MycN increases after NGF deprivation, for example, *ndrg1 *(Figures 5 and 6 and Table 2).

## Conclusions

The sympathetic neuron model is one of the best studied models of neuronal apoptosis. For the first time, we now have a global overview of the changes occurring at the transcriptional level in NGF-deprived sympathetic neurons. In the future, it will be interesting to determine how the regulated genes identified in this study contribute to the NGF withdrawal-induced death pathway. This may lead to the identification of new targets for the development of neuroprotective drugs that inhibit neuronal death following acute injuries to the nervous system or in neurodegenerative diseases.

## Methods

### Cell culture

Animal experiments were performed according to the Animals (Scientific Procedures) Act 1986 under a license reviewed and approved by the Biological Services Unit at University College London. Sympathetic neurons were isolated from the superior cervical ganglia (SCG) of 1-day-old Sprague Dawley rats (Biological Services Unit, University College London, UK) and cultured in Dulbecco's modified Eagle medium (DMEM; Life Technologies, USA) supplemented with 10% fetal calf serum (FCS), 2 mM glutamine (Invitrogen Ltd, UK) and penicillin-streptomycin as described previously (SCG medium; [61]). To limit the proliferation of non-neuronal cells, the antimitotic agents fluorodeoxyuridine and uridine (Sigma Aldrich) were added to the SCG medium at a final concentration of 20 μM. For some experiments, 2.5S NGF (Cedarlane, Canada) was also added to SCG medium at a final concentration of 50 ng/ml. Neurons were plated on 13 mm diameter glass coverslips coated with poly-*L*-lysine and laminin placed in 3.5 cm diameter dishes containing 2 ml of SCG medium and NGF for 5-7 days. In NGF withdrawal experiments, neurons were washed twice in SCG medium lacking NGF and then refed with SCG medium supplemented with a neutralising anti-NGF antibody at 100 ng/ml (Chemicon Europe Ltd, UK). The MLK inhibitor, CEP-11004 (Cephalon, Inc., USA) was dissolved in DMSO and used at a final concentration of 400 nM.

### RNA extraction

Total RNA was isolated from sympathetic neurons cultured for 7 days using an RNeasy mini kit (Qiagen). An on-column DNase digestion was performed to eliminate genomic DNA contamination using DNase I according to the manufacturer's instructions (Qiagen). RNA concentrations were determined using a NanoDrop spectrophotometer (ND-1000). RNA was further analysed for integrity and quality on an Agilent Bioanalyser.

### Array hybridisation

Up to 2 μg of total RNA was processed and labelled using the Affymetrix GeneChip Whole Transcript Sense Target Labelling Assay as outlined in the manufacturer's instructions. Hybridisation to Affymetrix Rat Exon 1.0 ST arrays was performed for 16 hours at 45°C with constant rotation. Exon array data are available from the ArrayExpress database (http://www.ebi.ac.uk/arrayexpress) under accession number E-MTAB-696.

### Analysis of array data

Signal estimates and normalisation for gene-level analysis were generated using the Probe Logarithmic Intensity Error Estimation (PLIER) algorithm implemented in the Expression Console software (Affymetrix). Only core, non cross-hybridising probe sets that map to well-annotated exons were included. To reduce noise, probe sets and transcript clusters which fell into the lowest quartile of the expression signal distribution across all samples were excluded from the dataset. Signal values were analysed using Bioconductor (R). Gene expression values were compared between the three sample groups (+NGF, -NGF and -NGF+CEP-11004) using the moderated t-statistic of the Bioconductor package, Limma [62]. To correct for multiple testing at the gene level, the Benjamini-Hochberg (FDR) test was applied to identify statistically significant differentially expressed genes (FDR adjusted p-values < 0.05). Lists of significantly up- and down-regulated genes obtained from statistical comparisons were subjected to functional enrichment analysis using DAVID annotation tools [63].

### Real-time quantitative PCR

Up to 1 μg of total RNA was reverse transcribed into cDNA using SuperScript II reverse transcriptase (Invitrogen) and oligo(dT) as described previously [64]. Ten nanograms of cDNA template was used for real time quantitative PCR using the ABI-Prism 7900HT fast Sequence Detection System (Applied Biosystems, Warrington, UK). *Txnip*, *ddit3*, *trib3, ndrg1 *and *mxi1 *mRNA levels were normalised to the level of the control genes, *gapdh *or *hprt1*. Pre-optimised Taqman^® ^*gapdh *(Rn99999916_s1), *hprt1 *(Mn00446968_m1), *txnip *(Rn01533885_g1), *trib3 *(Rn00595314_m1), *ddit3 *(Rn00492098_g1), *ndrg1 *(Rn01506130_m1), and *mxi1 *(Rn00565846_m1) primer/probe sets were supplied by Applied Biosystems. QPCR reactions were set up with 1 × primer/probe set and 1 × Taqman^® ^PCR Master Mix (Applied Biosystems). PCR conditions were 95°C for 20 seconds, followed by 40 cycles of 95°C for 1 second and 60°C for 20 seconds. QPCR data was analysed using the 2^-ΔΔCT ^relative quantitation method [65].

### Immunoblotting

Immunoblotting was performed as described previously [24]. Briefly, sympathetic neurons were harvested in 1 ml of ice-cold PBS, spun down and lysed in sample buffer (2% SDS, 2 mM β-mercaptoethanol, 60 mM Tris, pH 6.8, 0.01% bromophenol blue) for 10 minutes at 100°C. Proteins were separated on 12% SDS polyacrylamide gels and transferred to Immobilon-P (Millipore). After blocking for 45 min with 5% non-fat milk in TBS supplemented with 0.5% Tween-20, the membrane was incubated with different primary antibodies overnight at 4°C.

The following primary antibodies were used: rabbit polyclonal Trib3 antibody (M-165; Santa Cruz, USA), rabbit polyclonal Ndrg1 antibody (H-60; Santa Cruz), mouse monoclonal Txnip antibody (MBL International, Woburn, USA), mouse monoclonal CHOP10/Ddit3 antibody (B3; Santa Cruz), rabbit polyclonal Mxi1 antibody (G16; Santa Cruz), mouse monoclonal c-Jun antibody (BD Transduction Laboratories). Equivalent protein loading was confirmed by using a rabbit polyclonal ERK 1/2 antibody (Cell Signalling Technology).

### Immunofluorescence

Sympathetic neurons cultured on poly-*L*-lysine/laminin-coated glass coverslips were fixed using 4% paraformaldehyde at room temperature for 20 min, washed three times with PBS, and then permeabilised with 0.5% Triton X-100 in PBS at room temperature for 5 min. Neurons were then incubated in 50% normal goat serum in 1% BSA in PBS for 30 min at room temperature. After washing, neurons were incubated with primary antibody for 1 hour at room temperature, followed by a 45 min incubation with secondary antibody at room temperature. The following antibodies were used: mouse monoclonal phospho-c-Jun (ser63) antibody (Santa Cruz), rabbit polyclonal activated caspase-3 antibody (Abcam), mouse monoclonal cytochrome c antibody (BD Biosciences, Oxford, UK), rabbit polyclonal MAP2 antibody (Millipore). Fluoroscein- or rhodamine-conjugated goat anti-rabbit or anti-mouse secondary antibodies (Stratech, UK) were typically used at a dilution of 1:250. Neurons were rinsed in PBS and nuclei stained with DAPI dye in Antifade (DAKO) or Hoechst dye and mounted on glass slides. TUNEL staining was performed using an *in situ *cell death detection kit (Roche) according to the manufacturer's protocol.

### Microscopy and image collection

Slides were viewed on a Zeiss Axioplan 2 microscope using a Plan-Apochromat 63x/1.40 oil objective. Images were captured at room temperature (20°C) using a Quantix digital camera (Photometrics, USA) and SmartCapture VP software. For the different treatments for each gene, the optimal exposure time was determined using the +NGF (0 hour) coverslip and was kept constant for all subsequent images for the remaining timepoints. For immunofluorescence time course experiments, all coverslips in each series for a particular gene were analysed in parallel and then saved as TIFF files and viewed using Adobe Photoshop CS4. Brightfield images were collected using a Zeiss Axiovert 200 M microscope with a Plan-Apochromat 63x/1.40 oil objective. The microscope stage was maintained at 37°C with 5% CO_2_. Images were captured using a Zeiss axiocam and Axiovision 4.0 software.

### Statistical analysis

The statistical significance of differences between means was analysed by performing an unpaired Student's T-test (for two-tailed distributions). To compare normalised data to a control sample, that has no error associated to it (for example, where +NGF is set to 1), the log10 values of the data were taken and a one sample T-test was used (for two-tailed distributions) as previously described [57]. All data are presented as the mean ± S.E. of multiple experiments and significance is expressed as follows *P< 0.01.

## List of abbreviations

FDR: false discovery rate; GO: gene ontology; MLK: mixed lineage kinase; NGF: nerve growth factor; NS: not significant; PLIER: probe logarithmic intensity error estimation; ROS: reactive oxygen species; SCG: superior cervical ganglion; SE: standard error of the mean.

## Authors' contributions

RH carried out the real-time qPCR experiments. MK, FM and JH analysed and interpreted the microarray data. FM and MK performed the functional enrichment and gene ontology analysis and generated the heatmaps. MK performed all of the remaining experiments and analysis and wrote the manuscript. MH analysed the data and edited the manuscript. JH designed the study and wrote the manuscript. All authors read and approved the final manuscript

## Acknowledgements and Funding

We would like to thank Nipurna Jina for sample processing and quality control, Dr. Sonia Shah for help with the microarray analysis and Cephalon, Inc. for CEP-11004. We would also like to thank Dr. Solange Desagher for critical reading of this manuscript. This work was supported by a Wellcome Trust Senior Research Fellowship awarded to JH (Grant number 057700) and a Wellcome Trust VIP award to MK. FM was funded by the Annabel McEnery Children's Cancer Fund. We declare that there are no conflicts of interest.

## Supplementary Material

Additional file 1 **All genes up-regulated by NGF withdrawal in this study**. A PDF file listing the fold increase in RNA level after NGF withdrawal of all genes that are up-regulated more than 2-fold and have an FDR-adjusted p-value of < 0.01. Click here for file

Additional file 2 **All genes down-regulated by NGF withdrawal in this study**. A PDF file listing the fold decrease in RNA level after NGF withdrawal of all genes that are down-regulated more than 2-fold and have an FDR-adjusted p-value of < 0.01. Click here for file
