# Supplementary material for: Global analysis of gene expression in NGF-deprived sympathetic neurons identifies molecular pathways associated with cell death
Source: BMC Genomics. 2011 Nov 8;12:551. doi: 10.1186/1471-2164-12-551 (PMC3256215; doi:10.1186/1471-2164-12-551)
Supplement: Additional file 1 — All genes up-regulated by NGF withdrawal in this study. A PDF file listing the fold increase in RNA level after NGF withdrawal of all genes that are up-regulated more than 2-fold and have an FDR-adjusted p-value of < 0.01. [file 1471-2164-12-551-S1.PDF]

# Additional file 1

| ID | FC | P-value | Gene symbol |
|---|---|---|---|
| 7102680 | 9.78 | 2.62E-08 | Bid3 |
| 7120384 | 5.28 | 3.84E-07 | Pik3ip1 |
| 7246655 | 5.21 | 4.23E-06 | Trib3 |
| 7049929 | 4.72 | 8.80E-06 | Hamp |
| 7066011 | 4.69 | 5.29E-07 | Dusp1 |
| 7232394 | 4.35 | 2.16E-06 | Bcl2l11 |
| 7274003 | 4.29 | 7.01E-05 | Cga |
| 7087159 | 4.17 | 6.91E-07 | Pcp4 |
| 7315869 | 4.11 | 3.04E-06 | Ddit3 |
| 7197113 | 3.68 | 3.73E-06 | Txnip |
| 7111272 | 3.63 | 8.12E-06 | Btg2 |
| 7290196 | 3.58 | 2.16E-06 | Tspan1 |
| 7306585 | 3.41 | 3.21E-05 | Egln3 |
| 7210794 | 3.20 | 2.96E-06 | Efna1 |
| 7328494 | 3.18 | 3.28E-06 | Ndrg1 |
| 7162363 | 3.16 | 5.33E-05 | Gadd45g |
| 7045969 | 3.14 | 3.21E-05 | Cnksr3 |
| 7123310 | 3.10 | 6.01E-06 | Bmp3 |
| 7039439 | 3.05 | 9.28E-05 | Chrm1 |
| 7213318 | 2.95 | 2.16E-06 | Prg1 |
| 7297162 | 2.95 | 2.33E-06 | Slc26a3 |
| 7115455 | 2.89 | 3.55E-06 | Atf3 |
| 7299815 | 2.75 | 7.78E-06 | Smoc1 |
| 7132879 | 2.71 | 1.21E-05 | Stmn4 |
| 7347975 | 2.71 | 0.000205 | Filip1 |
| 7288744 | 2.62 | 4.17E-05 | Jun |
| 7156247 | 2.60 | 2.94E-05 | Atxn1 |
| 7294836 | 2.51 | 1.08E-05 | Rhoq |
| 7323193 | 2.51 | 0.000447 | Tcp11l2 |
| 7217331 | 2.50 | 5.70E-05 | Pim1 |
| 7188397 | 2.41 | 1.65E-05 | Tpo1 |
| 7073257 | 2.39 | 0.000322 | Map2k6 |
| 7219186 | 2.39 | 0.000809 | Pkib |
| 7254721 | 2.28 | 5.01E-05 | Eif2ak3 |
| 7227551 | 2.27 | 4.04E-05 | Kcnj3 |
| 7334980 | 2.27 | 0.00198 | Loh11cr2a |
| 7359661 | 2.25 | 0.000184 | Gtpbp2 |
| 7274595 | 2.23 | 0.00265 | Ccin |
| 7208971 | 2.23 | 0.00998 | P2ry14 |
| 7043189 | 2.22 | 8.98E-05 | Mxi1 |
| 7293932 | 2.17 | 1.17E-05 | Cntfr |
| 7182872 | 2.17 | 4.23E-05 | Herpud1 |
| 7095183 | 2.17 | 0.000225 | Klhl24 |
| 7052227 | 2.16 | 0.000112 | Chrna7 |
| 7087207 | 2.13 | 0.00102 | Mx2 |
| 7340763 | 2.10 | 0.000205 | Lrrc2 |
| 7379969 | 2.03 | 0.000188 | Wdr45 |
| 7257404 | 2.01 | 8.57E-05 | Il17re |

**Additional file 1: All genes up-regulated by NGF withdrawal in this study.** The fold increase in RNA level after NGF withdrawal (average of 3 independent experiments) is shown for all genes that are up-regulated more than 2-fold and have an FDR-adjusted p-value of <0.01. The level of gene expression in the presence of NGF was set to 1. ID, Affymetrix Transcript cluster ID; FC, Fold change.
