# Supplementary material for: Global analysis of gene expression in NGF-deprived sympathetic neurons identifies molecular pathways associated with cell death
Source: BMC Genomics. 2011 Nov 8;12:551. doi: 10.1186/1471-2164-12-551 (PMC3256215; doi:10.1186/1471-2164-12-551)
Supplement: Additional file 2 — All genes down-regulated by NGF withdrawal in this study. A PDF file listing the fold decrease in RNA level after NGF withdrawal of all genes that are down-regulated more than 2-fold and have an FDR-adjusted p-value of < 0.01. [file 1471-2164-12-551-S2.PDF]

Additional file 2

ID	FC	P-value	Gene symbol
7314286	-6.68	3.34E-07	Dusp6
7123848	-5.90	2.16E-06	Areg
7164422	-4.89	3.04E-06	Prl6a1
7166170	-4.86	2.05E-06	Idi1
7206103	-4.72	1.23E-05	Ca2
7113785	-4.72	2.10E-06	Hsd17b7
7260283	-4.69	4.26E-07	Insig1
7036421	-4.47	5.29E-07	Hs3st2
7133039	-4.35	1.18E-06	Stc1
7144691	-3.94	3.28E-06	Sc4mol
7057972	-3.46	5.47E-06	GalNAc4S6ST
7333634	-3.32	2.10E-06	Ldlr
7071108	-3.25	4.99E-06	unknown
7194074	-3.14	2.16E-06	Trpc4
7261367	-3.10	2.16E-06	Crot
7033513	-3.10	2.10E-06	Slc28a1
7232335	-2.91	1.11E-05	Adra2b
7094280	-2.89	1.85E-05	Bdh1
7089490	-2.81	2.54E-05	Dgkg
7202670	-2.79	5.87E-06	Hmgcr
7075416	-2.73	3.60E-05	Hn1l
7026698	-2.73	9.50E-06	Oprm1
7313174	-2.71	2.99E-05	Cry1
7336571	-2.69	9.50E-06	Ptpn9
7136761	-2.69	5.64E-06	Ptprg
7062917	-2.68	3.07E-05	Scd1
7266497	-2.68	2.25E-05	Hk2
7038734	-2.68	1.34E-05	Dhcr7
7317317	-2.66	3.04E-06	Sqle
7231641	-2.64	1.16E-05	Tyro3
7354355	-2.62	0.000188	Obfc2a
7348125	-2.60	6.20E-05	unknown
7138089	-2.60	3.08E-05	Homez
7101851	-2.60	2.16E-06	Aacs
7080099	-2.57	0.000101	Tmem97
7190101	-2.50	4.04E-05	Hmgcs1
7074098	-2.50	2.94E-05	Slc16a3
7080069	-2.48	0.00014	Tlcd1
7299483	-2.48	2.99E-05	Akap5
7164204	-2.45	0.000199	Agtr1a
7378456	-2.45	0.000103	Nsdhl
7356509	-2.43	2.83E-05	Acsl3
7343909	-2.43	2.15E-05	St3gal4
7097412	-2.43	3.73E-06	Vgf
7230218	-2.41	4.70E-05	Chst1
7273421	-2.39	2.94E-05	Hrpap20
7354636	-2.38	0.00195	Coq10b
7057305	-2.38	2.99E-05	Coro1a
7306041	-2.36	0.000184	Lrrn3
7344558	-2.36	5.01E-05	Sc5d
7186293	-2.36	4.23E-05	Mvd
7361215	-2.35	0.000308	Mgat4a
7257144	-2.35	3.05E-05	Bhlhb2
7277738	-2.35	5.61E-06	Ak3l1
7055029	-2.33	0.000655	P2ry6
7051330	-2.33	1.12E-05	Ptpn5
7350886	-2.30	0.000142	Scn5a
7361254	-2.28	0.000205	unknown
7188573	-2.28	3.37E-05	F2rl2
7250653	-2.28	3.08E-05	Cyp51
7112009	-2.23	0.000467	Rgs2
7098424	-2.23	0.000149	Lnk
7033786	-2.22	0.000525	Nox4
7305953	-2.22	0.000205	Arl4a
7182309	-2.20	9.36E-05	Pdp2
7249028	-2.20	2.94E-05	Rgs19
7304727	-2.19	0.000114	Mycn
7139220	-2.19	5.57E-05	Pnoc
7330860	-2.19	3.07E-05	Abcd2
7073725	-2.17	0.000149	Galr2
7236751	-2.17	3.05E-05	Tubb2c
7081403	-2.16	0.000336	Pctp
7353158	-2.16	7.68E-05	Khdrbs2
7296550	-2.14	0.00278	Nol10
7169197	-2.13	7.20E-05	Egr1
7379299	-2.11	0.00238	Chst7
7139070	-2.10	0.000119	Fdft1
7240420	-2.10	4.04E-05	Pde11a
7188693	-2.10	2.04E-05	Enc1
7148281	-2.08	0.000866	unknown
7178411	-2.08	2.94E-05	unknown
7085814	-2.07	9.65E-05	Robo1
7287531	-2.06	0.00022	Dbccr1
7353812	-2.06	0.000199	Il1r1
7232988	-2.04	0.000475	unknown
7097915	-2.03	0.000218	unknown

Additional file 2: All genes down-regulated by NGF withdrawal in this study. The fold decrease in RNA level after NGF withdrawal (average of 3 independent experiments) is shown for all genes that are down-regulated more than 2-fold and have an FDR-adjusted p-value of <0.01. The level of gene expression in the presence of NGF was set to 1. ID, Affymetrix Transcript cluster ID; FC, Fold change. Transcript cluster IDs with no associated annotation are listed as unknown.
